# Supplementary material for: Dichotomy between in-plane magnetic susceptibility and resistivity anisotropies in extremely strained BaFe2As2
Source: Nat Commun. 2017 Sep 11;8:504. doi: 10.1038/s41467-017-00712-3 (PMC5593886; doi:10.1038/s41467-017-00712-3)
Supplement: Supplementary file 1 — Supplementary Information [file 41467_2017_712_MOESM1_ESM.pdf]

File Name: Supplementary Information
Description: Supplementary Figures, Supplementary Notes and Supplementary References

File Name: Peer Review File

Supplementary Note 1 In-plane spin anisotropy of the stripe antiferromagnetic phase

The itinerant electron system of the parent iron-based superconductors can be described by a multi-orbital Hubbard Hamiltonian, which consists of the non-interacting hopping Hamiltonian within the $3d$-orbital manifold, H_0, and the Hubbard-Hund interaction, $H_{\rm int}$,

$$H = H_0 + H_{\rm int}, \tag{1}$$

with

$$H_0 = \sum_\sigma \sum_{i,j} \sum_{\mu,\nu} c^\dagger_{i\mu\sigma} \left(t^{\mu\nu}_{ij} - \mu_0 \delta_{ij} \delta_{\mu\nu} \right) c_{j\nu\sigma}, \tag{2}$$

and

$$H_{\rm int} = U \sum_{i,\mu} n_{i\mu\uparrow} n_{i\mu\downarrow} + U' \sum_{i,\mu<\nu,\sigma} n_{i\mu\sigma} n_{i\nu\bar\sigma} + (U' - J) \sum_{i,\mu<\nu,\sigma} n_{i\mu\sigma} n_{i\nu\sigma} +$$
$$J \sum_{i,\mu<\nu,\sigma} c^\dagger_{i\mu\sigma} c^\dagger_{i\nu\bar\sigma} c_{i\mu\bar\sigma} c_{i\nu\sigma} + J' \sum_{i,\mu<\nu,\sigma} c^\dagger_{i\mu\sigma} c^\dagger_{i\mu\bar\sigma} c_{i\nu\bar\sigma} c_{i\nu\sigma}. \tag{3}$$

The indices $\mu, \nu \in \{d_{xz}, d_{yz}, d_{x^2-y^2}, d_{xy}, d_{3z^2-r^2}\}$ specify the $3d$-Fe orbitals and i, j run over the sites of the square lattice. The doping is fixed by the chemical potential μ_0. The interactions are parametrized by an intra-orbital on-site Hubbard-U, an inter-orbital coupling U', Hund's coupling J and pair hopping J'. We employ $U' = U - 2J$, $J = J'$ and set $J = U/4$. The fermionic operators $c^\dagger_{i\mu\sigma}$, $c_{i\mu\sigma}$ are the creation and annihilation operators, respectively. We specify the hopping parameters $t^{\mu\nu}_{ij}$ according to the band structure obtained by Ikeda *et al.* [1] or Daghofer et al. [2, 3] for the five or three orbital models, respectively.

The approximate nesting between hole pockets around Γ and M and electron pockets around X and Y promotes strong fluctuations in the particle-hole channel at wave vectors $\mathbf{Q}_1 = (\pi, 0)$ and $\mathbf{Q}_2 = (0, \pi)$. The electronic states at the Fermi level are dominated by the d_{xz}, d_{yz} and d_{xy} orbitals [1, 2], as shown in Supplementary Figure 1. While the hole-pockets centered around Γ are formed by d_{xz} and d_{yz} orbitals, the hole pocket at M is mainly of d_{xy} character. The electron pockets at X and Y feature a mixed orbital character, where the inner part facing towards the BZ center is d_{xy} dominated, and the outer parts are d_{yz} and d_{xz} dominated around $(\pi, 0)$ and $(0, \pi)$, respectively. This allows for the further reduction of the 5-orbital model to the three-orbital model only [2].

This directionality of the SDW order parameter, on top of the breaking of rotational symmetry in spin space, also breaks the C_4 symmetry of the five orbital model down to a C_2 symmetry. The two different, orbitally resolved SDW order parameters read as $\mathbf{M}^{\mu\nu}_1 = \frac{1}{\mathcal{N}} \sum_{\mathbf{k},\sigma,\sigma'} \langle c^\dagger_{\mathbf{k}+\mathbf{Q}_1\mu\sigma} \boldsymbol{\sigma}_{\sigma\sigma'} c_{\mathbf{k}\nu\sigma'} \rangle$, and $\mathbf{M}^{\mu\nu}_2 = \frac{1}{\mathcal{N}} \sum_{\mathbf{k},\sigma,\sigma'} \langle c^\dagger_{\mathbf{k}+\mathbf{Q}_2\mu\sigma} \boldsymbol{\sigma}_{\sigma\sigma'} c_{\mathbf{k}\nu\sigma'} \rangle$, with $\mathcal{N}$ the number of unit cells and $\boldsymbol{\sigma}$ the vector of Pauli matrices. Here, the fermionic creation and annihilation operators for orbital Bloch states with wave vector $\mathbf{k}$ are defined as $c^\dagger_{\mathbf{k}\mu\sigma} = \frac{1}{\sqrt{\mathcal{N}}} \sum_i \mathrm{e}^{-\mathrm{i}\mathbf{k}\cdot\mathbf{r}_i} c^\dagger_{i\mu\sigma}$, $c_{\mathbf{k}\mu\sigma} = \frac{1}{\sqrt{\mathcal{N}}} \sum_i \mathrm{e}^{\mathrm{i}\mathbf{k}\cdot\mathbf{r}_i} c_{i\mu\sigma}$. Taking the orbital trace yields the magnetic moments $\mathbf{M}_1$, $\mathbf{M}_2$ of the two SDW configurations.

Besides the band dispersions, the non-interacting Hamiltonian must also contain the SOC term $\lambda \mathbf{S} \cdot \mathbf{L}$, with $\mathbf{S}$ denoting the spin angular momentum operator and $\mathbf{L}$, the orbital angular momentum operator, projected from the $L = 2$ cubic harmonic basis to the orbital basis [4]. Furthermore, in the following we assume that the system possesses a striped AF order with $\mathbf{Q}_1$ ordering wave-vector and the magnetic moment is pointing along the ordering momentum, i.e. x-direction. Such an order appears to be the ground state for zero doping in several studies of the typical models of the iron-based superconductors [4, 5]. To understand the origin of anisotropy in the uniform susceptibility in the magnetic state, we note that the magnetic inter-orbital components of the mean-field magnetizations for the C_2 phase were found to be negligible compared to the intra-orbital terms [5]. Furthermore, as mentioned above there are three orbitals contributing to the Fermi surfaces, however, only one of them has a significant portion of the intra-orbital nesting.

As a result the magnetization for the striped antiferromagnetic state with $\mathbf{Q}_1$ wave vector has largest contribution that arises from the yz-orbital, as shown in Supplementary Figure 1.

Next we compute the components of the magnetic susceptibility $\chi_0^{xx/yy/zz}$ in the multi-orbital case,

$$\chi_0^{uu}(\mathbf{q}, \omega) = -\sum_{\mathbf{k}ij} \eta^{uu}(i, \mathbf{k}; j, \mathbf{k}+\mathbf{q}) \times \frac{f(E_j(\mathbf{k}+\mathbf{q})) - f(E_i(\mathbf{k}))}{E_j(\mathbf{k}+\mathbf{q}) - E_i(\mathbf{k}) + \omega + i0^+}, \quad u = x, y, z, \tag{4}$$

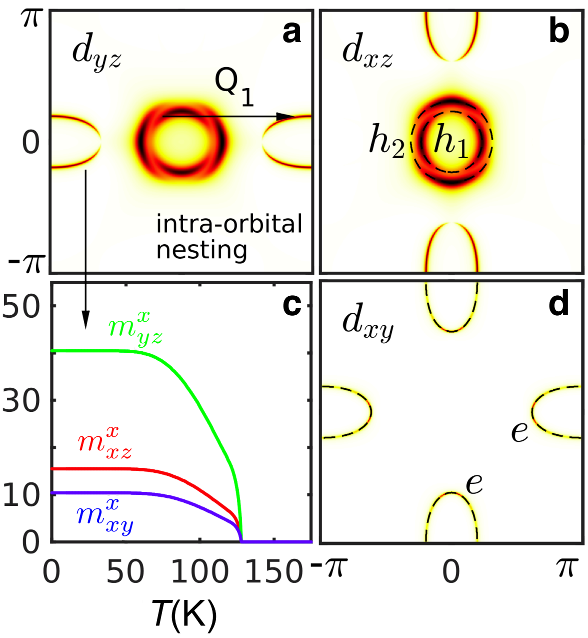

Supplementary Figure 1. **Typical distribution of the orbital content at the Fermi surface for the iron-based compounds** Contributions from (a)d_{yz}, (b)d_{xz}, (d)d_{xy} orbitals at the Fermi surfaces. (a) For the wave-vector $\mathbf{Q}_1$, the intra-orbital nesting of the d_{yz}-orbitals dominates over the other orbitals contributions. As a direct consequence, the SDW gap (c) and the magnetic Umklapp susceptibility will acquire the largest contribution due the d_{yz}-orbital. The magnetization of the other two orbitals, present at the Fermi level, is mainly induced by the Hund coupling J. Here, m^i_l corresponds to the magnetization of the $l = yz, xz, xy$ orbital along the spin orientation $i = x, y, z$.

which includes the tensor

$$[\eta^{uv}(i,\mathbf{k};j,\mathbf{k}+\mathbf{q})]^{s\gamma,t\delta}_{q\beta,p\alpha} = \sigma^u_{\alpha\beta}\sigma^v_{\gamma\delta} \times a_{q\beta}(i,\mathbf{k})a^*_{s\gamma}(i,\mathbf{k})a_{t\delta}(j,\mathbf{k}+\mathbf{q})a^*_{p\alpha}(j,\mathbf{k}+\mathbf{q}), \quad (5)$$

expressed with the help of the Pauli matrices σ^u and the the elements of the unitary transformations from the band to the orbital basis, a. The physical susceptibility is then obtained by taking the trace over $p = q$ and $s = t$ orbitals.

It is straightforward to show that the anisotropy of the susceptibility enters through the orbital-dressing factors (5) and presence of the Umklapp terms in the antiferromagnetic translational-symmetry broken state. We find for the bare susceptibility:

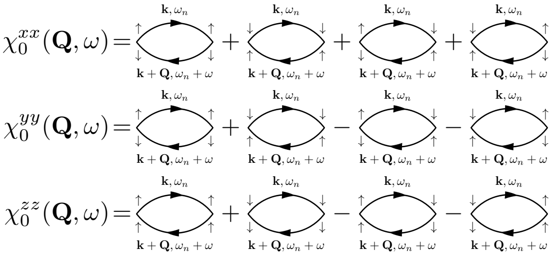

(6)

Note that in the paramagnetic state and for vanishing spin-orbit coupling, the first two bubbles of each component are equal while the last two Umklapp terms vanish, ensuring the overall $O(3)$ symmetry of the system. If spin-orbit coupling is finite, we find $\chi^{yy} > \chi^{xx,zz}$ at $\mathbf{Q}_1 = (\pi, 0)$ so that an alignment of the magnetic moments parallel to $\mathbf{Q}_1$ is favored ($\mathrm{M}^x \neq 0$, $\mathrm{M}^{y,z} = 0$).

Although spin-orbit coupling is large enough to lower the symmetry by favoring M^x over M^y for the AF wave-vector $\mathbf{Q}_1$, it is not large enough to account for the size of the in-plane anisotropy observed below the AF transition temperature. In the following we show that the in-plane splitting of the uniform susceptibility is caused by the intra-orbital Umklapp terms of the yz orbital (xz respectively for $\mathbf{Q}_2$). In particular, looking at the Eq.(6), one sees

that the difference of the two in-plane components originates from the third and fourth bubble diagrams due to the different sign for χ^{xx} and χ^{yy},

$$G_{yz,\sigma;yz,\bar{\sigma}}(i,\mathbf{k})G_{yz,\sigma;yz,\bar{\sigma}}(j,\mathbf{k}+\mathbf{q}+\mathbf{Q}_1). \tag{7}$$

Evaluating the sums we find for the splitting

$$[\chi_0^{xx}-\chi_0^{yy}]_{(\mathbf{q}=0)} = 4\Big[(m_{yz}^x)^2-(m_{yz}^y)^2\Big]\sum_{i\mathbf{k}}|a_{yz\uparrow}(h_i,\mathbf{k})|^4|a_{yz\downarrow}(e,\mathbf{k}+\mathbf{Q}_1)|^4\ \frac{f(E_i(\mathbf{k}))-f(E_e(\mathbf{k}+\mathbf{Q}_1))}{\big[E_i(\mathbf{k})-E_e(\mathbf{k}+\mathbf{Q}_1)\big]^3} \tag{8}$$

where we have set $\omega=0$ and denote m_l^i to be the magnetization of the $l=yz,xz,xy$ orbital along the spin orientation $i=x,y,z$. As one clearly sees in the stripe AF phase with ordering momentum $\mathbf{Q}_1$ with spins aligned either parallel or antiparallel to the x-direction ($\mathrm{M}^x\neq 0, m_{yz}^x\neq 0$), both transverse components of the susceptibility split and one has $\chi_0^{yy}(\mathbf{q}=0,\omega=0)>\chi_0^{xx}(\mathbf{q}=0,\omega=0)$. Furthermore, the sign of the anisotropy is reversed ($\mathrm{M}^y\neq 0, m_{yz}^y\neq 0$) if the moments would be pointing out perpendicular to the ordering wave vector. In simple terms the largest AF gap in the spin subspace reduces the corresponding component of the uniform susceptibility.

Our analytical results for the yz orbitals are fully confirmed by the full numerical calculations using the realistic tight-binding models [1, 2]. In particular, in Figure 4 we show the calculated uniform susceptibility splitting calculated within random phase approximation. In addition, the numerical study confirms that the in-plane anisotropy is determined by the Umklapp susceptibility involving yz and that the sign of the anisotropy depends on the orientation of the magnetic moments.

Finally we note by passing that the origin of the magnetic anisotropy in the uniform susceptibility cannot be due to the simple ferro-orbital ordering ($n_{xz}-n_{yz}\neq 0$), introduced by the structural (nematic) transition at $T_\mathrm{S}>T_\mathrm{N}$ although it breaks the anisotropy between the x and the y component of the spin susceptibility. One finds in this case that the splitting will be proportional to $\Delta_{\mathrm{oo}}\lambda^2$ and thus its sign is reversed as compared to the effect of the magnetic ordering. The magnitude of the orbital order has not been chosen arbitrarily. In particular, in the striped antiferromagnetic state with $\mathbf{Q}_1=(\pi,0)$ ordering wave-vector this value of Δ_{oo} appears naturally as a result of the self-consistent mean-field calculations of the dominant magnetic order due to lowering the crystal symmetry of the lattice from the tetragonal (C_4) to the orthorhombic one (C_2) in the antiferromagnetic state. The degeneracy between the xz and yz orbitals is lifted at the Γ-point in the C_2 phase, which yields for the parameters of the model Δ_{oo} =-25 meV. To test the effect of the orbital nematic order on the $\chi_0(\mathbf{q}=0,\omega=0)$ alone, i.e. without a magnetic order, we simply set magnetic order to zero but keep Δ_{oo} as before. Obviously the results demonstrate that in ferropnictides the Δ_{oo} alone cannot explain the experimental data without invoking magnetic degrees of freedom. This value of Δ_{oo} also matches that observed by ARPES within a factor of two [6].

Supplementary Note 2 In-plane uniform magnetic susceptibility anisotropy of a second sample

In Supplementary Figure 2, the anisotropic in-plane susceptibility measured on a second sample are presented, which shows very similar behavior as that of sample #1 shown in Figure 3.

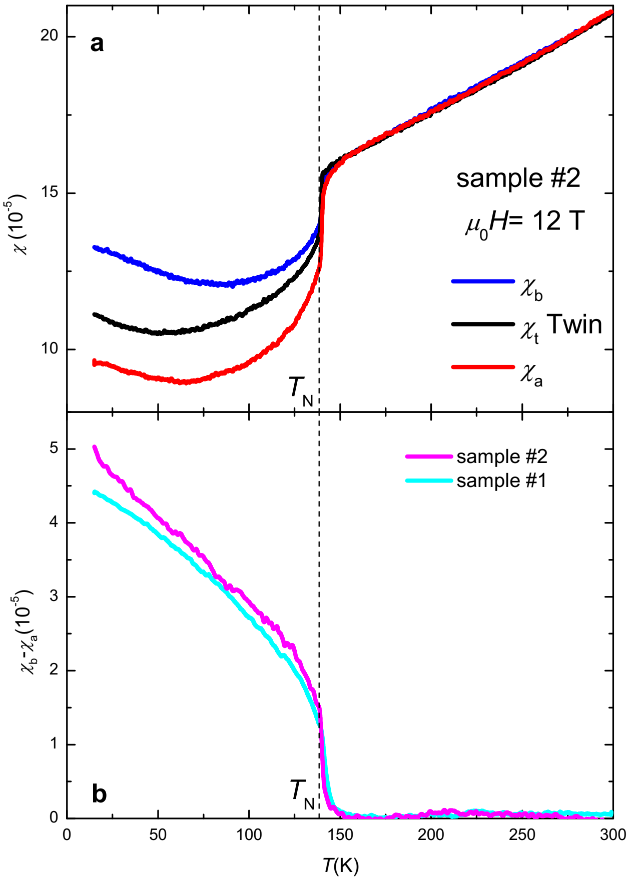

Supplementary Figure 2. **Susceptibility anisotropy of different samples** (a) Anisotropic in-plane susceptibilities of a second sample. (b) Comparison of the in-plane susceptibility anisotropy obtained from two cystals. The data of sample #1 is also shown in the Figure 3 of main text.

Supplementary References

[1] Ikeda, H., Arita, R. & Kuneš, J. Phase diagram and gap anisotropy in iron-pnictide superconductors. *Phys. Rev. B* **81**, 054502 (2010).

[2] Daghofer, M., Nicholson, A., Moreo, A. & Dagotto, E. Three orbital model for the iron-based superconductors. *Phys. Rev. B* **81**, 014511 (2010).

[3] Scherer, D. D., Eremin, I. & Andersen, B. M. Collective magnetic excitations of C_4-symmetric magnetic states in iron-based superconductors. *Phys. Rev. B* **94**, 180405 (2016).

[4] Christensen, M. H., Kang, J., Andersen, B. M., Eremin, I. & Fernandes, R. M. Spin reorientation driven by the interplay between spin-orbit coupling and Hund's rule coupling in iron pnictides. *Phys. Rev. B* **92**, 214509 (2015).

[5] Gastiasoro, M. N., Paul, I., Wang, Y., Hirschfeld, P. J. & Andersen, B. M. Emergent defect states as a source of resistivity anisotropy in the nematic phase of iron pnictides. *Phys. Rev. Lett.* **113**, 127001 (2014).

[6] Yi, M. *et al.* Symmetry-breaking orbital anisotropy observed for detwinned $Ba(Fe_{1-x}Co_x)_2As_2$ above the spin density wave transition. *Proc. Natl. Acad. Sci. U.S.A.* **108**, 6878–6883 (2011).
